# Observations on the Application of Sutures in Severely-Contused Lacerated Wounds

**Published:** 1822-06

**Authors:** J. C. Yeatman


### Art. II.—*Observations on the Application of Sutures in severely-contused Lacerated Wounds.* By J. C. Yeatman, Esq.

THE scientific and practical surgeon will smile at my giving myself the trouble, in the beginning of the nineteenth century, to offer strictures on the practice of applying sutures in the above description of wounds; imagining, doubtless, that the practice is extinct, except in certain wounds of the abdomen, throat, and face. The great doctrines of inflammation, as laid down by the ingenious and indefatigable John Hunter, and as taught in the modern schools of surgery, one would have supposed, would have effectually banished the practice of applying sutures in such wounds, and have given rise, in every instance of this sort, to a milder mode of treatment,—a mode of treatment more consonant with the improved state of surgery.

Severely-contused lacerated wounds must, from the very nature of their infliction, be subjected to a higher degree of inflammation than is requisite to establish the adhesive process: they frequently terminate in suppuration, and sometimes in gangrene. Living animal matter, torn asunder and bruised, must suffer in proportion to the impulse given to the moving power with which the injury is inflicted, and the degree of resistance with which that impulse is met. It is not, however, easy, *a priori*, to judge whether any, or how much, of the injured soft parts will unite by the first intention; and therefore, where this practice is attempted, the treatment should be of the mildest kind, and best calculated to moderate inflammation. Hence, after freeing extraneous matters from the wound, and placing the majority of the muscles, connected with the parts injured, in the most relaxed state, slips of adhesive plaster are applied, to bring the torn edges as near together as the nature of the wound will admit of; and a soft pledget, a pad of linen, and a roller, wetted in one of the cold sedative lotions, are applied; and, further to obviate that inflammation which is so liable, in these cases, to lead to suppuration, leeches to the neighbourhood of the injured parts, with general bleeding, diaphoretics, and saline purgatives, are had recourse to:—in short, the antiphlogistic treatment is enjoined. On the other hand, where the soft parts have received so much injury, that their vitality is destroyed, the beer-grounds fermenting poultice, opium, a generous diet, &c. are indicated. Suppuration is now to be established, and the wound healed by granulations.

Such is the general outline of the nature and treatment of severely-contused lacerated wounds, in reference to adhesion, suppuration, and gangrene.

There are cases in which the soft parts may possess different degrees of vitality, particularly near the site of old wounds and ulcers, and have sustained different degrees of injury. Such cases require a mixed kind of treatment, for which no rules can be laid down, and which that surgeon alone can regulate who is well acquainted with pathology and the principles of his profession.

Thus much have I said, by way of introduction.

I shall now call the serious attention of practitioners, who are unfortunately tainted with the notions of the old school, or who are not well *grounded* in the knowledge of their profession, to an error in practice, which, notwithstanding all that has been said against it, they are frequently committing,—viz. the practice of applying sutures to contused lacerated wounds of the extremities, with the view to effect union by the first intention; as though living animal matter were to be kept on the stretch, like Indian-rubber or fiddle-strings, and be obedient only to the laws of mechanics!

Case I.—A young healthy man severely bruised and lacerated the integuments and muscles, immediately above the patella, which were brought together by sutures, the application of adhesive slips, and a roller. In a few hours great inflammation came on; and, although the stiches, &c. were removed, an emollient poultice applied, and saline purgatives and diaphoretics given, yet the parts mortified and sloughed to a considerable extent. Suppuration was established, and the surgeon obliged to heal the wound by the tedious process of granulation; whereas, a different mode of treatment would have insured union by the first intention.

Case II.—A robust young man lacerated the fore-arm to about five inches in length, beginning an inch above the inner condyle of the humerus. The extensor carpi ulnaris and pronator radii teres were torn asunder, and part of the interosseal ligament exposed to view. Whilst the surgeon was amusing himself with a quotation from Butler,—

> So learned Taliacotius, from
> The brawny part of porter's bum,
> Cut supplemental noses, which
> Would last as long as parent breech;
> But, when the date of Nock was out,
> Off dropp'd the sympathetic snout,—

he sewed the lacerated muscles together, took up a branch of the recurrens ulnaris, implanted sutures in the external wound, applied adhesive slips and bandage firmly over them, and sent the patient to bed. The next morning the youth died. On removing the roller and sticking-plaster, the parts were found in a gangrenous state.

Case III.—John Curtis, æt. forty-five; in stature, very robust; in habit, choleric and sanguineous. October 17, 1816, fell from the top of a large heap of fern into a saw-pit, lacerating the thigh by a ragged piece of timber lying across its mouth. The patient lay at the bottom of the pit from twelve o'clock at night till six the next morning, when he contrived, by the assistance of a person, to crawl out of the saw-pit, and to hobble to a public-house; whence he was conveyed, after being dressed by a surgeon, to the poor-house, in a sedan-chair, by order of the overseer.

18*th*.—Saw Curtis this afternoon; found him in great pain; removed a roller and the slips of adhesive-plaster, which had been firmly applied by a surgeon, as also ten interrupted sutures, which had been inserted to keep the integuments in their situation, and which had caused so much inflammation, that the swoln soft parts were bursting them asunder. Situation of the wound, the anterior and middle part of the thigh; extent, five inches; shape, triangular; nature, a considerable portion of the integuments forming a flap; its base towards the groin, and apex pointing to the patella; portions of the rectus femoris and vastus internus exposed to view, by the retraction of the flap. On removing the stiches, an emollient poultice was applied, the patient bled, and a saline purgative given.

*19th.*—Pulse quick, full, and strong; tongue furred; thirst great; inflammation extending upwards to the inguin, and downwards to the patella. Bled him a second time; repeated the purgative, and ordered a grain of opium twice a-day.

*Evening.*—The whole flap becoming livid.

*20th.*—A line of separation between the dead and living parts, formed across the base of the torn flap; inflammation on the decline; pain subsided; pulse soft and irregular; tongue covered with brown fur. Ordered the strong beer-grounds fermenting poultice, bark, and opium.

*21st.*—Nearly as yesterday, except that the line of separation is more distinct.

*22d.*—Torn flap, separating; surrounding tension and inflammation still less.

*23d.*—Flap came away with the poultice.

In this case, luckily, the mortification was prevented from extending beyond the flap, by the removal of the sutures and the antiphlogistic treatment, or it is impossible to say how far and deep it might have gone, and what might have been the issue of the case. As it was, the wound was obliged to be healed by the slow process of suppuration and granulation, as in the first case; and, the vitality of the parts having been weakened, the cicatrix, to this day, is apt to break out into a sore on receiving the slightest injury.

*Frome, Somerset; April 5th,* 1822.

